# Feasibility of Quality of Life Assessment in Patients with Lymphoma Aged ≥80 Years Receiving Reduced-Intensity Chemotherapy: A Single-Institute Study

**DOI:** 10.3390/hematolrep16010001

**Published:** 2023-12-22

**Authors:** Satoshi Yamasaki

**Affiliations:** 1 Department of Hematology and Clinical Research Institute, National Hospital Organization Kyushu Medical Center, Fukuoka 810-0065, Japan; yamasaki.satoshi.668@m.kyushu-u.ac.jp or yamas009@gmail.com; Tel.: +81-977-27-1600; Fax: +81-977-27-1641; 2 Department of Internal Medicine, Kyushu University Beppu Hospital, 4546 Tsurumihara, Tsurumi, Beppu 874-0838, Japan

**Keywords:** older patients, lymphoma, reduced-intensity chemotherapy, quality of life, comprehensive geriatric assessment

## Abstract

Quality of life (QOL) must be carefully monitored in older patients with lymphoma who are suitable for chemotherapy, but few reports have assessed QOL in older patients who received reduced-intensity chemotherapy. This study investigated QOL in patients with lymphoma aged ≥80 years to clarify the feasibility of such assessments following reduced-intensity chemotherapy. QOL was prospectively analyzed (using the QOL Questionnaire for Cancer Patients Treated with Anticancer Drugs (QOL-ACD)] and the SF-36^®^, a comprehensive survey of patient health) among 13 patients (seven women) aged ≥80 years with lymphoma who received reduced-intensity chemotherapy at 4-week intervals at Kyushu University Beppu Hospital between June 2022 and August 2023. Patients were assessed at baseline, in the middle of the protocol, at the end of the protocol, and 6 months after the end of the protocol. The overall response rate was 69%. Almost all severe adverse events (10 patients) occurred during early cycles (cycles 1–2). Common adverse events included hematological toxicities such as neutropenia (10 patients). The daily activity (*p* = 0.048) and social attitude (*p* = 0.027) scores of the QOL-ACD and the general health perception (*p* = 0.044) and social functioning (*p* = 0.030) scores of the SF-36^®^ were significantly improved during and after chemotherapy. Reduced-dose chemotherapy, if implemented before treatment selection, might permit evaluations of QOL in older patients aged ≥80 years; further investigation is warranted.

## 1. Introduction

According to 2021 data obtained from the Cancer Registry and Statistics, Cancer Information Service, National Cancer Center, Japan (http://gdb.ganjoho.jp/graph_db/index?lang=ja (accessed on 19 September 2023)), the incidence rates of lymphoma were estimated at 69.7 and 38.8 per 100,000 persons in men and women aged 80–84 years, respectively, and 112.6 and 61.3 per 100,000 persons in men and women aged ≥85 years, respectively. 

The measurement of quality of life (QOL) in lymphoma provides valuable information on health status and treatment effects that cannot be obtained using other methods [1]. In addition, baseline QOL could be a prognostic predictor, as previously described [2,3], and it could facilitate patient–physician communication, thereby promoting shared decision making [4]. Patients with a higher risk of cancer tend to experience more symptoms of anxiety and distress during the screening process [5], but there are few reports on QOL in patients aged ≥80 years with lymphoma receiving reduced-intensity chemotherapy, such as mini-CHOP (cyclophosphamide [CPA], adriamycin [ADR], vincristine [VCR], and prednisolone [PSL]) plus rituximab (RTX) for patients with diffuse large B-cell lymphoma (DLBCL) [6]. There are no randomized controlled trials of reduced-intensity chemotherapy in patients aged ≥80 years with lymphoma. Appropriate reduced-intensity chemotherapies have not been established, and only two phase II trials for DLBCL have been reported [7,8].

Comprehensive geriatric assessments (CGAs) have been used to help identify older patients with lymphoma who are suitable for standard-dose chemotherapy [9], and the American Society of Clinical Oncology suggested that a CGA should be considered for older patients receiving chemotherapy [10]. However, there are few reports on the use of a CGA, which might be an effective tool for guiding therapeutic strategies for patients aged ≥80 years with lymphoma who received reduced-intensity chemotherapy [11].

It is believed that evaluating QOL in older patients is more difficult than in younger patients. In addition, it is unclear whether QOL can be evaluated in patients aged ≥80 years, who are ineligible for most clinical trials. To fill the evidence gap in real-world experience of the management of older patients, I conducted a prospective investigation at Kyushu University Beppu Hospital. This study investigated the feasibility of QOL assessment among patients aged ≥80 years with lymphoma who received reduced-intensity chemotherapy.

## 2. Patients and Methods

### 2.1. Study Oversight

This study was performed in accordance with institutional guidelines and the principles of the Declaration of Helsinki. This prospective observational study was approved by the ethics boards of Kyushu University Hospital, Japan (No. 22164-00), and written informed consent was provided by all participants at Kyushu University Beppu Hospital between June 2022 and August 2023.

### 2.2. Patient Selection

The inclusion criteria were as follows: (1) newly diagnosed and histologically proven de novo DLBCL, mantle cell lymphoma (MCL), angioimmunoblastic T cell lymphoma (AITL), or adult T cell leukemia/lymphoma (ATLL) according to the World Health Organization classification [12]; (2) age ≥ 80 years at the time of diagnosis; and (3) the receipt of at least one cycle of mini-CHP (CPA, ADR, and PSL) therapy, polatuzumab vedotin (Pola) plus RTX, bortezomib and rituximab (VR), or brentuximab vedotin (BV) or mini-CHOP plus mogamulizumab (MOG) at 4-week intervals in a first-line setting. The exclusion criteria were as follows: (1) a history of dementia (because of the difficulty of performing a CGA); (2) history of a previous hematological malignancy; and (3) central nervous system involvement.

The examined variables were as follows: (1) patient-related variables including age at diagnosis, sex, Eastern Cooperative Oncology Group performance status, Geriatric 8 (G8) score [13], instrumental activities of daily living (IADL) score [14], and the Charlson comorbidity index (CCI) [15]; (2) disease-related variables including Ann Arbor stage, bone marrow and extranodal involvement, bulky disease (>7.5 cm), serum lactate dehydrogenase elevation, serum albumin, hemoglobin, beta-2-microglobulin, soluble interleukin-2 receptor, and the International Prognostic Index [16]; and (3) outcome variables, including the number of initial chemotherapies, radiotherapy, the overall response rate (ORR, complete remission (CR) plus partial remission (PR)), and relapse or disease progression after treatment. The enrolled patients were followed up with until death or the end of the follow-up period (the minimum duration of follow-up was 6 months after the final chemotherapy cycle). 

### 2.3. Treatment

All patients received Pola-RTX-, VR-, or BV-mini-CHP (400 mg/m^2^ CPA and 25 mg/m^2^ ADR intravenously on day 1 and 40 mg/m^2^ PSL orally or intravenously) or MOG-mini-CHOP (400 mg/m^2^ CPA, 1 mg/body VCR, and 25 mg/m^2^ ADR intravenously on day 1 and 40 mg/m^2^ PSL orally or intravenously). Patients received 1.8 mg/kg Pola or 1.3 mg/m^2^ bortezomib plus 375 mg/m^2^ RTX, 1.8 mg/kg BV, or 1 mg/kg MOG. Dose modification and the timing of the start of subsequent cycles were determined at the physician’s discretion. In patients with grade ≥3 adverse events (AEs) according to the National Cancer Institute Common Toxicity Criteria version 4.0, during treatment, the dose of each chemotherapeutic drug in the subsequent cycle was reduced, and the protocol regimen (0%, 25%, 50%, 75%, or 100% dose) was delayed at the physician’s discretion.

### 2.4. Efficacy Evaluation

Physical examination, computed tomography (CT), and bone marrow analysis were used for disease status assessments. To evaluate the tumor response, the Revised Response Criteria for Malignant Lymphoma [17] using positron emission tomography (PET)-CT was selected. CT scans of the neck, thorax, abdomen, and pelvis and PET-CT scans were performed every 3 months for up to 24 months or until the initiation of alternative chemotherapy, whichever occurred first. The CT and PET-CT scans were performed by experienced radiologists.

The QOL Questionnaire for Cancer Patients Treated with Anticancer Drugs (QOL-ACD), a QOL measurement tool specifically for patients with cancer, and the SF-36^®^, a comprehensive survey measuring patient health, were used to assess QOL. Patients were assessed at baseline, in the middle of treatment, at the end of the protocol, and 6 months after the end of the protocol. A statistically significant change in a QOL score when compared with the baseline score was considered a clinically meaningful change.

### 2.5. Statistical Methods

The frequencies and descriptive statistics, disease variables, and outcome variables in patients with lymphoma were analyzed. Continuous variables were expressed as medians and ranges. Categorical variables were expressed as numbers and percentages. AEs from the start of treatment until 6 months after the completion of treatment were graded according to the National Cancer Institute Common Toxicity Criteria version 4.0. Overall survival (OS) was defined as the time from diagnosis to death from any cause. Patients who had not relapsed, progressed, or died were censored at the date of the last follow-up. OS was calculated using the Kaplan–Meier method and compared using the log-rank test. Regarding QOL-ACD and SF-36 scores, changes from baseline were analyzed using the Wilcoxon signed-rank test. All tests were one-sided, and *p* < 0.05 was considered statistically significant. Analyses were conducted using EZR (Saitama Medical Center, Saitama, Japan; http://www.jichi.ac.jp/saitama-sct/SaitamaHP.files/statmedEN.html (accessed on 19 September 2023)) [18], which is a graphical user interface for R (The R Foundation for Statistical Computing, version 4.2.2; www.r-project.org (accessed on 19 September 2023)), and a modified version of R commander (version 2.8-0) designed to add statistical functions. 

## 3. Results

### 3.1. Patient Characteristics

**Figure 1:**
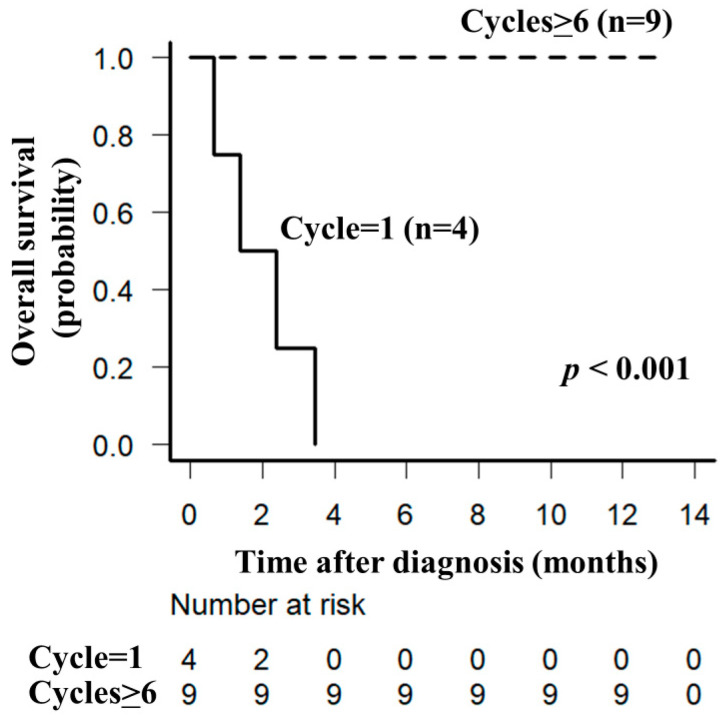
Overall survival in patients with lymphoma is associated with the number of cycles of chemotherapy. Overall survival in 13 patients was plotted using the Kaplan–Meier method. Overall survival significantly differed between the completion of 1 and ≥6 cycles of chemotherapy.

Overall, 13 of the 30 enrolled patients with DLBCL (*n* = 4), MCL (*n* = 3), AITL (*n* = 3), or ATLL (*n* = 3) met the inclusion criteria. Meanwhile, two enrolled patients (one man aged 90 years and one woman aged 92 years) did not receive any chemotherapies, and 15 enrolled patients (five men and ten women aged 82–97 years) could not undergo a QOL assessment because of dementia. The baseline characteristics of the participants are presented in Table 1. Five patients (38%) were older than 85 years. In all patients, the G8 score was <10, the IADL score was 2, and the CCI was ≥2. All patients were negative for human immunodeficiency virus infection. Among the thirteen patients, nine (69%) patients received ≥6 cycles of chemotherapy. The ORR was 69% (CR, 38%; PR, 31%). The main cause of death was lymphoma progression. The median follow-up period was 7 (range, 1–8) months, and four (31%) patients (one man with DLBCL and one woman each with DLBC, MCL, and AITL) died of lymphoma (Figure 1).

### 3.2. Toxicity Assessments

Table 2 shows all AEs including grade 3 and 4 events. During early cycles (cycles 1–2), almost all severe AEs occurred. Common AEs included hematological toxicities such as neutropenia, which improved with supportive treatment. There were no treatment-related mortalities. Although every patient received granulocyte colony-stimulating factor (G-CSF), 10 (77%) patients displayed decreased white blood cell and neutrophil counts during only cycle 1 or both cycles 1 and 2. Peripheral sensory neuropathy occurred in three (39%) patients after the last chemotherapy cycle.

### 3.3. QOL

**Figure 2:**
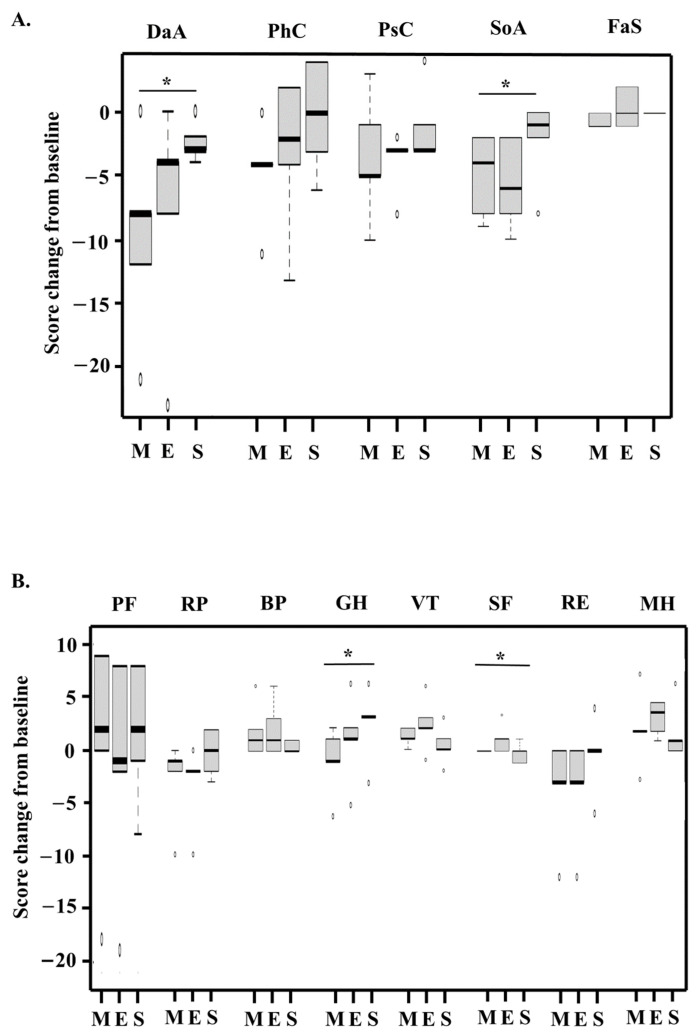
Box and whisker plots of total quality of life (QOL) scores using the QOL-ACD (**A**) and the SF-36 health survey (**B**). The bottom and top of the box are the 25th and 75th percentiles, respectively. The thick band is the 50th percentile (median). The ends of the whiskers represent the lowest datum still within 1.5 of the interquartile range (IQR) of the lower quartile and the highest datum still within 1.5 of the IQR of the upper quartile. The open circles are outliers between 1.5 and 3 of the IQR from the end of a box. QOL was assessed at baseline, during the middle of chemotherapy (M), at the end of chemotherapy (E), and 6 months after the end of chemotherapy (S). * A statistically significant change in a QOL score compared among the scores for M, E, and S. BP, bodily pain; DaA, daily activity; FaS, face scale; GH, general health perception; MH, general mental health; PF, physical functioning; PhC, physical condition; PsC, psychological condition; RE, role of limitations caused by personal or emotional health problems; RP, role of limitations caused by health problems; SF, social functioning; SoA, social attitude; VT, vitality.

Changes in QOL from baseline are presented in Figure 2A,B. Using the QOL-ACD, significant improvements in daily activity (*p* = 0.048) and social attitude (*p* = 0.027) scores were recorded in the middle and at the end of chemotherapy and 6 months after the last chemotherapy cycle. Regarding the SF-36 health survey, general health perception (*p* = 0.044) and social functioning (*p* = 0.030) scores were significantly improved in the middle and at the end of chemotherapy and 6 months after the last chemotherapy cycle compared with the baseline status.

## 4. Discussion

To the best of my knowledge, this is the first study to evaluate QOL after Pola-RTX-mini-CHP, VR-mini-CHP, BV-mini-CHP, or MOG-mini-CHOP for patients with lymphoma aged ≥80 years. This study demonstrated that reduced-dose chemotherapy, if implemented before treatment selection, might permit QOL assessment in patients aged ≥80 years.

My group reported that conducting a CGA, especially the IADL before initial chemotherapy, might be helpful for identifying patients suitable for R-mini-CHOP at 4-week intervals among patients with DLBCL aged ≥80 years [19]. I found that a CGA did not have a dependent association with OS, contradicting prior research. There are several reasons that can explain this discrepancy. First, in this study, the results of the CGA were extremely poor in all patients, for example, G8 < 10, IADL = 2, and CCI ≥ 2. Another reason is that these data could not help in conducting a CGA to select suitable patients for reduced-intensity chemotherapy due to various lymphomas and chemotherapy.

The main findings of this study for the QOL-ACD were significantly better daily activity and social attitude scores for patients in the middle and at the end of chemotherapy and 6 months after the last chemotherapy cycle versus the baseline values (before initial chemotherapy). Regarding the SF-36 health survey, the general health perception and social functioning scores were significantly better in the middle and at the end of chemotherapy and 6 months after the last chemotherapy cycle than at baseline. Although a CGA was hypothesized to improve QOL, this study did not observe an effect on QOL. However, decreases in functional status affect QOL as such decreases are related to decreases in activity, potentially resulting in worse QOL [20]. Prior research suggested that a CGA can reduce treatment-associated toxicity [21].

The results of this study indicate that reduced-intensity chemotherapy provides a good balance between disease control and toxicities in the short term and represents a manageable treatment option in patients aged ≥80 years aiming for cure. Bataillard et al. reviewed survival outcomes following reduced-intensity R-CHOP in patients with DLBCL and showed that in patients aged ≥80 years, the use of reduced-intensity chemotherapy compared with standard-dose chemotherapy did not consistently affect survival [22]. The reduced incidences of neutropenia, anemia, or febrile neutropenia after reduced-intensity chemotherapy in patients aged ≥80 years resulted in better clinical outcomes; however, most AEs occurred during the first two cycles. This difference can be explained by the fact that all patients were treated as inpatients for a large part of the treatment period to ensure their safety, and they received intensive supportive care with prophylaxis including G-CSF. Careful and intensive management at a hospital enables elderly patients to achieve sufficient outcomes, but some issues, including the cost of treatment, remain to be resolved. This suggests that reduced-intensity chemotherapy with intensive supportive care, especially during the first two cycles, should be considered for older patients who cannot tolerate standard-dose chemotherapy. Arcari et al. summarized the therapeutic options for patients aged ≥65 years with DLBCL, noting that patients who are unfit and/or frail frequently need reduced-intensity chemotherapy or the substitution of particular medications with less toxic ones [23]. If efficacy and safety data are confirmed in future clinical trials, a chemotherapy-free approach could become an option for offering a curative treatment even in frail patients, including patients aged ≥80 years. Rozental et al. discussed the use of novel agents in older patients with MCL, noting that reduced-intensity treatment has been shown to be efficacious in the older population, with reduced toxicity profiles [24]. Recently, there is a trend toward chemotherapy-free regimens, targeted therapies such as BTK, BCL-2, and PI3K inhibitors, and immunotherapies such as lenalidomide, bispecific antibody, and chimeric antigen receptor T-cells which could provide a promising strategy for older patients. Given these multiple potentially non-cross-reactive mechanisms, studies of rationally designed combination strategies hold promise for improving outcomes and possibly curing patients aged ≥80 years with lymphoma.

Aging is a complex natural process that progressively leads to a loss of physiological integrity with organ dysfunction, increased inflammation, and susceptibility to genetic damage and epigenetic modification. Some patients did not receive standard-dose chemotherapy due to their own preference or that of the physician. Therefore, they did not receive chemotherapy strictly because of age or comorbidities, which might have induced a bias, possibly exemplified by older age and poor outcome risks, as previously reported [19]. Although the current study was a prospective study, which usually has fewer potential sources of bias and confounding than a retrospective study, there was a possibility of selection bias regarding ineligibility for standard-dose chemotherapy because no proper randomization could be achieved.

This prospective study had several limitations, including its single-institutional nature. First, this study could not clarify the relationship between the CGA and QOL. Second, the number of obtained samples was smaller than the minimum number identified in the sample size calculation (*n* = 30) because 17 enrolled patients did not meet the inclusion criteria. Third, carrying out a QOL measurement in older patients is difficult because of dementia. Fourth, the research samples were mostly obtained from lymphoma survivors whose QOL might have been better, which, in turn, influenced the results of the analysis. Fifth, the results of the final analysis only identified two factors related to QOL; this could be attributable to differences in the instruments used in previous studies, or a larger number of samples might be required to achieve statistically significant results. In addition, B and T cell lymphomas have vastly different biology and treatment trajectories, and it is not ideal to compare the outcomes of vastly different diseases with varied prognoses and vastly different chemotherapy regimens in the same study. Furthermore, a decrease in treatment intensity might have resulted in poorer treatment outcomes in patients aged ≥80 years. The effects of different salvage therapies were unknown because of the range of available salvage therapies for lymphoma, and the salvage therapies after relapsed or refractory disease were selected according to physician and patient preferences. Finally, the sample size; selection bias, especially relating to the exclusion of patients with a history of dementia because of the difficulty of performing a CGA and measuring QOL; and the short follow-up duration might have limited my ability to analyze the patient outcomes after initial chemotherapy.

In conclusion, reduced-dose chemotherapy, if implemented before treatment selection, might permit evaluations of QOL in patients aged ≥80 years. The relationship between CGA and QOL requires further evaluation in large-scale prospective studies using patient-oriented outcomes, and the results of such studies could facilitate shared decision-making.

## Figures and Tables

**Table 1:** Characteristics of patients with lymphoma.

Characteristic		DLBCL	MCL	AITL	ATLL
		*n* = 4	*n* = 3	*n* = 3	*n* = 3
Median age (range), years		84 (80–93)	84 (80–88)	87 (82–90)	81 (80–82)
>85 years old, *n* (%)		2 (50)	1 (25)	2 (67)	0
Sex, *n* (%)	male	2 (50)	2 (67)	1 (33)	1 (33)
	female	2 (50)	1 (33)	2 (67)	2 (67)
ECOG PS, *n* (%)	1	2 (50)	2 (67)	1 (33)	2 (67)
	2	2 (50)	1 (33)	1 (33)	1 (33)
	3	0	0	1 (33)	0
G8, median (range)		7 (7–9)	8 (6–9)	6 (4–8)	8 (7–9)
IADL, median (range)		2	2	2	2
CCI, median (range)		2 (2–3)	3 (2–4)	3 (2–3)	2 (2–4)
Ann Arbor stage IV at diagnosis, *n* (%)		4 (100)	3 (100)	3 (100)	3 (100)
BM involvement, *n* (%)		2 (50)	3 (100)	3 (100)	3 (100)
LDH elevation, *n* (%)		4 (100)	3 (100)	3 (100)	3 (100)
ALB, median (range), g/dL		2.9 (2.5–3.2)	3.0 (2.3–3.2)	2.6 (2.4–2.8)	2.4 (2.2–2.5)
ALB >2.8 g/dL		3 (75)	2 (67)	1 (33)	0
Hemoglobin, median (range), g/dL		10.3 (6.2–12.5)	9.5 (7.2–11.2)	8.2 (5.8–10.2)	7.8 (6.1–9.8)
Hemoglobin < 12 g/dL		3 (75)	3 (100)	3 (100)	3 (100)
β2M, median (range), mg/L		2.7 (2.5–3.0)	3.2 (2.4–3.5)	3.2 (2.4–3.6)	3.0 (2.5–3.5)
β2M > 3.0 mg/L		1 (25)	2 (67)	2 (67)	2 (67)
sIL-2R, median (range), ×10^3^ U/mL		1.9 (1.5–3.2)	4.5 (3.5–5.6)	1.2 (1.1–1.3)	23 (11–25)
IPI, *n* (%)	Low (0–1)	1 (25)	2 (67)	0	1 (33)
	Intermediate (2–3)	3 (75)	1 (33)	3 (100)	2 (67)
No. of cycles of initial chemotherapy, median (range)		3 (1–6)	6 (1–8)	6 (1–8)	6 (6–8)
≥6 cycles, *n* (%)		2 (50)	2 (67)	2 (67)	3 (100)
ORR, *n* (%)		2 (50)	2 (67)	2 (67)	3 (100)
Relapse/progression, *n* (%)		2 (50)	1 (33)	1 (33)	0
Death due to lymphoma, *n* (%)		2 (50)	1 (33)	1 (33)	0
Median (range) follow-up, months		7 (1–13)	12 (3–13)	12 (1–13)	13 (12–13)

DLBCL, diffuse large B cell lymphoma; MCL, mantle cell lymphoma; AITL, angioimmunoblastic T cell lymphoma; ATLL, adult T cell leukemia/lymphoma; ECOG PS, Eastern Cooperative Oncology Group performance status; G8, Geriatric 8; IADL, instrumental activities of daily living; CCI, Charlson Comorbidity Index; BM, bone marrow; LDH, lactate dehydrogenase; ALB, serum albumin; β2M, beta-2-microglobulin; sIL-2R, soluble interleukin-2 receptor; IPI, International Prognostic Index; ORR, overall response rate; PFS progression-free survival; CI, confidence interval; OS, overall survival.

**Table 2:** Grade ≥ 3 adverse events attributable to reduced-intensity chemotherapy.

Toxicity	DLBCL	MCL	AITL	ATLL
	*n* = 4	*n* = 4	*n* = 3	*n* = 3
Hematological toxicities, *n* (%)				
WBC count decreased	2 (50)	2 (50)	3 (100)	3 (100)
Neutrophil count decreased	2 (50)	2 (50)	3 (100)	3 (100)
Lymphocyte count decreased	0	1 (25)	1 (33)	3 (100)
Anemia	2 (50)	2 (50)	2 (67)	3 (100)
Infections, *n* (%)				
Febrile neutropenia	2 (50)	2 (50)	1 (33)	3 (100)
Pneumonia	0	0	1 (25)	1 (25)
Non-hematological toxicities, *n* (%)				
Gastrointestinal				
Anorexia	0	0	1 (33)	1 (33)
Nausea	0	0	1 (33)	1 (33)
Neurological				
Peripheral sensory neuropathy	0	1 (25)	1 (33)	1 (33)

DLBCL, diffuse large B-cell lymphoma; MCL, mantle cell lymphoma; AITL, angioimmunoblastic T cell lymphoma; ATLL, adult T cell leukemia/lymphoma; WBC, white blood cell.

## Data Availability

The institutional review board of Kyushu University Hospital, Japan, does not allow open access. However, upon reasonable request, additional analyses can be performed after contacting the corresponding author.
